# Characterization of Shiga toxin-producing *Escherichia coli* in raw beef from informal and commercial abattoirs

**DOI:** 10.1371/journal.pone.0243828

**Published:** 2020-12-17

**Authors:** Kaarina N. Nehoya, Ndinomholo Hamatui, Renatus P. Shilangale, Harris Onywera, Jeya Kennedy, Lamech M. Mwapagha

**Affiliations:** 1 Department of Health Sciences, Faculty of Health and Applied Sciences, Namibia University of Science and Technology, Windhoek, Namibia; 2 Directorate of Veterinary Services, Ministry of Agriculture Water and Forestry, Windhoek, Namibia; 3 Central Veterinary Laboratory, Ministry of Agriculture Water and Forestry, Windhoek, Namibia; 4 Institute of Infectious Disease and Molecular Medicine, University of Cape Town, Cape Town, South Africa; 5 Division of Medical Virology, Department of Pathology, Faculty of Health Sciences, University of Cape Town, Cape Town, South Africa; 6 Department of Natural and Applied Sciences, Faculty of Health and Applied Sciences, Namibia University of Science and Technology, Windhoek, Namibia; CINVESTAV-IPN, MEXICO

## Abstract

Shiga toxin-producing *Escherichia coli* are foodborne pathogens that are mostly associated with beef products and have been implicated in human illness. *E*.*coli*-associated illness range from asymptomatic conditions of mild diarrhoea to haemorrhagic colitis which can progress into life threatening haemolytic uremic syndrome (HUS). Beef from cattle are regarded as the main reservoir of Shiga toxin-producing *E*. *coli* (STEC) pathogen. The aim of this study was to assess the level and sources of contamination of raw beef with STEC, and determine the incidences of STEC strains in raw beef from informal and commercial abattoirs in Windhoek, Namibia. A total of 204 raw beef samples, 37 equipment and 29 hand swabs were collected and tested for STEC. The meat samples were first enriched with pre-warmed buffered peptone water, cultured on Tryptone Bile X-Glucuronide and CHROMagar STEC, and then sub-cultured on nutrient agar. The presence of *E*.*coli* in the samples was confirmed by using VITEK 2 *E*.*coli* identification cards and PCR. The overall prevalence of STEC in the meat samples from both the abattoirs was 41.66% raw beef samples; 5.40% equipment swabs; and none of the hand swabs was STEC positive. From the STEC positive meat samples 29.41% contained one of the major STEC strains. Moreover, 52% of the 25 samples that contained the major STECs were characterised by *eae* and *stx*_*1*_, 8% characterised by *eae* and *stx*_*2*_ while 40% were characterised by *eae*, *stx*_*1*_ and *stx*_*2*_ virulence genes. This study has revealed the necessity for proper training on meat safety (for meat handlers) as well as the development, implementation and maintenance of effective sanitary dressing procedures at abattoirs to eliminate beef contamination by STECs thereby ensuring the production of wholesome meat, and to prevent the occurrences of STEC infections.

## Introduction

Approximately 2,801,001 acute illness, 3,890 cases of haemolytic uremic syndrome (HUS), 270 cases of permanent end-stage renal disease (ESRD) and 230 deaths in human are caused by Shiga toxin-producing *Escherichia coli* (STEC) globally [1]. STEC are a group of foodborne pathogenic *Escherichia coli* strains that produce cytotoxins with potential to cause severe enteric and systemic health conditions in humans [2]. Diseases caused by STEC range from uncomplicated diarrhoea to bloody diarrhoea and haemorrhagic colitis (HC) which often progress into HUS [3].

The most important transmission route for STEC is through the consumption of contaminated raw or undercooked ground beef [4]. *E*. *coli* O157:H7 has been identified as one of the STEC strains responsible for severe foodborne illness and morbidity worldwide [6]. Of late, it has become more clearly that six specific non-O157 STECs serogroups namely, O26, O45, O103, O111, O121 and O145 have been causing foodborne diseases comparable in severity to those caused by *E*. *coli* O157:H7 [5].

Cattle are considered primary reservoirs for STEC and, in general, they do not show pathological symptoms caused by these bacteria [6, 7]. However, equipment, particularly knives, saws and tables used in the beef carcass production line where good hygienic practices (GHP) is not maintained can further become a vector to spread STEC onto other carcasses and cuts of meat [8].

Due to high demand of beef worldwide and lack of proper hygienic practices several studies have shown an upsurge of STEC epidemics linked to human sickness globally [9–11]. In Africa, STEC related diarrheal outbreak associated with poor hygiene and the consumption of contaminated food have been reported in a several countries, including Namibia, South Africa, Eswatini, Kenya, Nigeria, Ivory Coast and in the Democratic Republic of Congo [3, 12–15].

In Namibia, there are limited data on the occurrence of STEC strains in beef from informal and commercial abattoirs as well as in human and environmental samples [15]. Despite the known severity of STEC illness in humans, very few studies have been carried out in Namibia regarding the presence of STEC in meat [3]. Some studies have shown that interventions implemented to control STEC at some local abattoirs have failed to significantly reduce the contamination of beef by STEC [16]. These limitations are of great concern to the field of public health, specifically to meat hygiene, disease prevention and control. Thus, this study aimed to determine STEC contamination of raw beef in informal and commercial slaughterhouses in Windhoek, Namibia.

## Methodology

### Background of the study area

Study was carried out in Windhoek the capital city of Namibia, located in the country’s highlands, of the Khomas region. There are two informal abattoirs and one large commercial abattoir that slaughter cattle. In this study, the aforementioned abattoirs are identified as Abattoir A that slaughters ∼200–350 cattle per day (commercial abattoir), Abattoir B that slaughters ∼50–70 cattle per day and Abattoir C that slaughters ∼10–35 cattle per day (informal abattoirs). Abattoir A, is a beef processing plant located in the Khomas region, abattoir B, is located 30 Km north of the Khomas region and abattoir C, is located 5 Kilometres north of the Khomas region.

### Study design

This was a quantitative, descriptive cross-sectional study design undertaken to determine the contamination level of raw beef with *E*. *coli* O157:H7 and the six top non-O157 STEC strains, to establish the sources of contamination and to investigate the incidences of STEC in raw beef at informal and commercial abattoirs in Windhoek. Samples were collected five times a week from July—November 2018.

### Sample size

The sample size was determined using the formula of a known population outlined by Kothari [17]. A total number of 270 samples were collected from abattoirs. These included 204 meat samples and 66 equipment and hand swabs (Table 1).

**Table 1:** Summary of sample numbers from both informal and commercial abattoirs.

Type of abattoir	Abattoir name	No. of samples		
		Meat	Equipment	Hand Swabs
Commercial abattoir	Abattoir A	100	23	19
Informal abattoir	Abattoir B	76	7	6
	Abattoir C	28	7	4
Total		204	37	29

### Sample collection

#### Meat samples

Simple random sampling based on N60 method was applied to collect meat samples from beef carcasses from the commercial and informal abattoirs in Windhoek [18]. Two 5 carcass composite samples (325g and 375g each) collected per lot each day over five months from ten arbitrarily selected carcasses. Twelve surface slices, of which the total is sixty, were pooled from different parts of each of the five carcasses including chuck, navel plate, flank brisket and fore-shank to represent one sample. A total number of 104 raw beef samples were collected from informal abattoirs, while one hundred beef samples were collected from the commercial abattoir. These were then transported to the laboratory for *E*. *coli* O157:H7 and the six top non-O157 STEC strains analysis.

**Fig 1:**
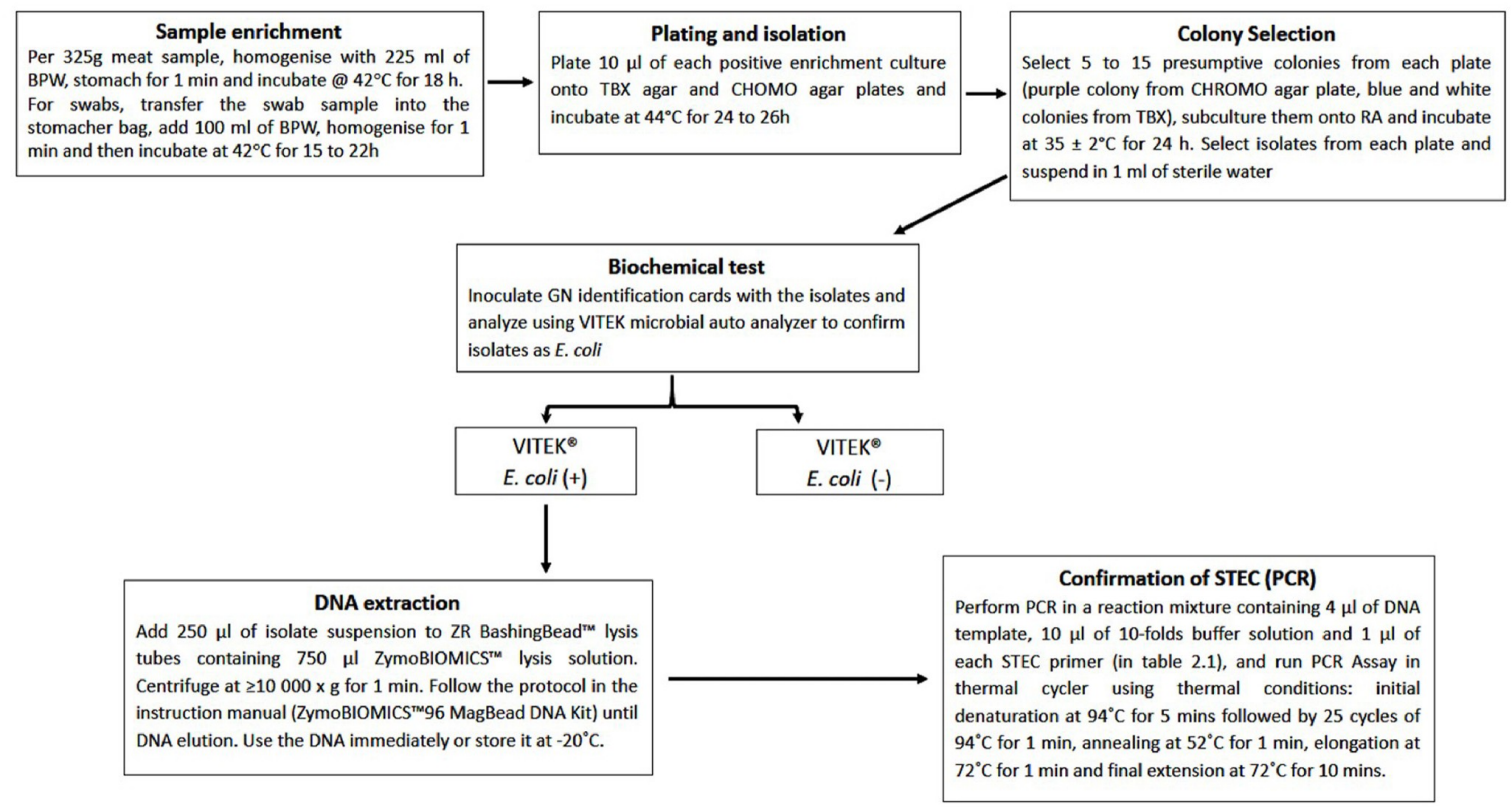
Summary of sample analysis. All the samples were first enriched then cultured on both TBX and CHROMagar plates. Biochemical test was then done on positive bacterial colonies and PCR was used to confirm STEC done on the positive presumptive bacterial samples using specific primers.

A total of fifty-three (n = 66) equipment and naked hand swab samples were collected from both commercial and informal abattoirs. These were collected aseptically, using sterile stick swabs by rubbing firmly over the predetermined surface area using a 10 cm^2^ template. Each swab sample was labelled immediately after sampling and was packed in a cool box with ice packs and transported to Central Veterinary lab for STEC analysis (Fig 1).

#### Equipment swabs

Swabs (n = 37) were taken from the knives used at both the skinning and evisceration stations before the commencement of the slaughtering process as well as during the production operations. This was repeated throughout the production process for three weeks.

#### Hand swabs

Hand swabs (n = 29) were collected from the employees at both the carcass skinning and evisceration stations. As for equipment swabs, two hand swab samples were collected before skinning and evisceration began (after the workers have washed their hands and ready to skin and eviscerate carcases) and then another two hand swabs were collected during the skinning and evisceration of carcasses.

### Laboratory analysis

#### Sample enrichment

Approximately 325g of meat sample was homogenised with 975 ml of pre-warmed (42°C) of buffered peptone water (BPW) in a stomacher machine (stomacher^®^ 3500 circulator machine) at 200 rpm for 2 minutes. The enrichments were incubated at 37°C for 18–24 hours. One positive control (canned beef spiked with 10μl of STEC reference colonies, either STEC O26 serotype or O103 serotype) and one blank negative control (canned beef) were also prepared and incubated, the same way as the samples. The positive control was employed for confirmation while the negative control was for prepared to ensure that there was no contamination during the analysis. Each swab sample was transferred into the stomacher bag respectively. 100ml of BPW was added to the swab in the stomacher bag, homogenised for 2 minutes and incubated at 37°C for 18–24 hours. One positive control (swab sample spiked with either STEC O26 serotype or O103 serotype) and one negative control (sterile swab) were also prepared.

#### Culturing of samples and isolation of pure colonies

For each sample, 10μl was taken and streaked onto Tryptone bile x-glucuronide (TBX) agar plates and on CHROMagar STEC plates respectively by the use of sterile wire loops. TBX agar plates were incubated at 44°C for 18–24 hours while CHROMagar STEC plates were incubated at 37°C for 18–24 hours. Following incubation, 5–10 colonies (blue-green colonies on TBX plates, mauve colonies on CHROMagar STEC plates) were selected from each plate and sub-cultured onto nutrient agar plates respectively, to get pure cultures. The plates were incubated at 37°C for 18 to 48 hours.

#### Biochemical confirmation test

The biochemical confirmation test for *E*.*coli* was performed using a VITEK^®^ 2 system according to the manufacturer’s instructions [19]. On the other hand, three presumptive STEC bacteria colonies (50-100mg) from nutrient agar plates were suspended in 200 μl of double distilled water (for DNA extraction) and 200 μl of nutrient broth in Eppendorf tubes (for preservation, at -80°C). DNA extraction was done on the suspended bacterial colonies.

#### DNA extraction and quantification

DNA was extracted from the presumptive STEC colonies on CHROMagar using Zymo Research DNA extraction kit, *Quick*-DNA^™^ Fungal/Bacterial Miniprep Kit (D6005) according to manufacturer’s instruction. The DNA was then quantified using a NanoDrop spectrophotometer (NanoDrop one, Thermo Scientific).

#### PCR analysis and confirmation

DNA analysis was done on STEC virulence genes, intimin (*eae*), shiga toxin 1 (*stx*_*1*_*)* and shiga toxin 2 (*stx*_*2*_*)*, and the top six STECs (O26, O45, O103, O111, O121, O145) plus *E*. *coli* O157:H7 (fliCh7, rfbE) (Table 2). PCR (MegaCycler^™^ Thermal Cycler Machine—Edvotek^™^ 542) was done using the Novataq master mix kit (71007–3) in a reaction volume of 20 μl. All amplifications were performed as follows: initial denaturation at 94°C for 4 min, followed by 30 cycles at 94°C for30s, and 55°C for 30s, 72°C for 1min and 72°C for 5 min. PCR results were resolved using 1.5% agarose gel containing 0.5 μg of ethidium bromide per ml of gel solution from stock solution (10 mg/ml), run for 1 hour at 100 volts and viewed under UV light.

**Table 2:** List of primer sequences against which the samples have been tested.

Target Genes	Primer	Primer sequence (5’– 3’)	Annealing temperature (°C)	Amplicon size (bp)	Reference
*stx*_*1*_	stx1-F	TGTCGCATAGTGGAACCTCA	52	655	[20]
	Stx1-R	TGCGCACTGAGAAGAAGAGA			
*stx*_*2*_	stx2-F	CCATGACAACGGACAAGCAGTT	52	477	[20]
	stx2-R	TGTCGCCAGTTATCTGACATTC			
*eae*	eae-F	CATTATGGAACGGCAGAGGT	50	375	[20]
	eae-R	ACGGATATCGAAGCCATTTG			
*wzx*026	026F	AGGGTGCGAATGCCATATT	50	417	[21]
	026R	GACATAATGACATACCACGAGCA			
*wzx*045	045-F	GGGCTGTCCAGACAGTTCAT	50	890	[21]
	045-R	TGTACTGCACCAATGCACCT			
*wzx*103	0103F	GCAGAAAATCAAGGTGATTACG	50	740	[21]
	0103R	GGTTAAAGCCATGCTCAACG			
*wzx*0111	0111F	TGCATCTTCATTATCACACCAC	50	230	[21]
	0111R	ACCGCAAATGCGATAATAACA			
*wbq*0121	0121F	TCAGCAGAGTGGAACTAATTTTGT	50	587	[21]
	0121R	TGAGCACTAGATGAAAAGTATGGCT			
*wzx*0145	0145F	TCAAGTGTTGGATTAAGAGGGATT	50	523	[21]
	0145R	CACTCGCGGACACAGTACC			
*fliCh7*	fliCh7F	GCGCTGTCGAGTTCTATCGAGC	60	625	[22]
	fliCh7R	CAACGGTGACTTTATCGCCATTCC			
*rfbE*	rfbEF	CAGGAGAAGGTGGAATGGTTGTC	60	296	[23]
	rfbER	TTAGAATTGAGACCATCCAATAAG			

### Ethical consideration

Ethical clearance to conduct the study was given by all the relevant bodies i.e. NUST, Ministry of Agriculture Water and Forestry, City of Windhoek and abattoirs” and written informed consent was given by the respective abattoir employees for the collection of hand swab samples.

### Statistical analysis

All experiments were performed in triplicate unless stated otherwise. The statistical analysis of the variables under study, was performed using Chi-square independence test and the Z test to compare two proportions. Statistical significance was regarded at a p value <0.05 [24].

## Results

### Contamination level of raw beef with specific STEC strains

**Fig 2:**
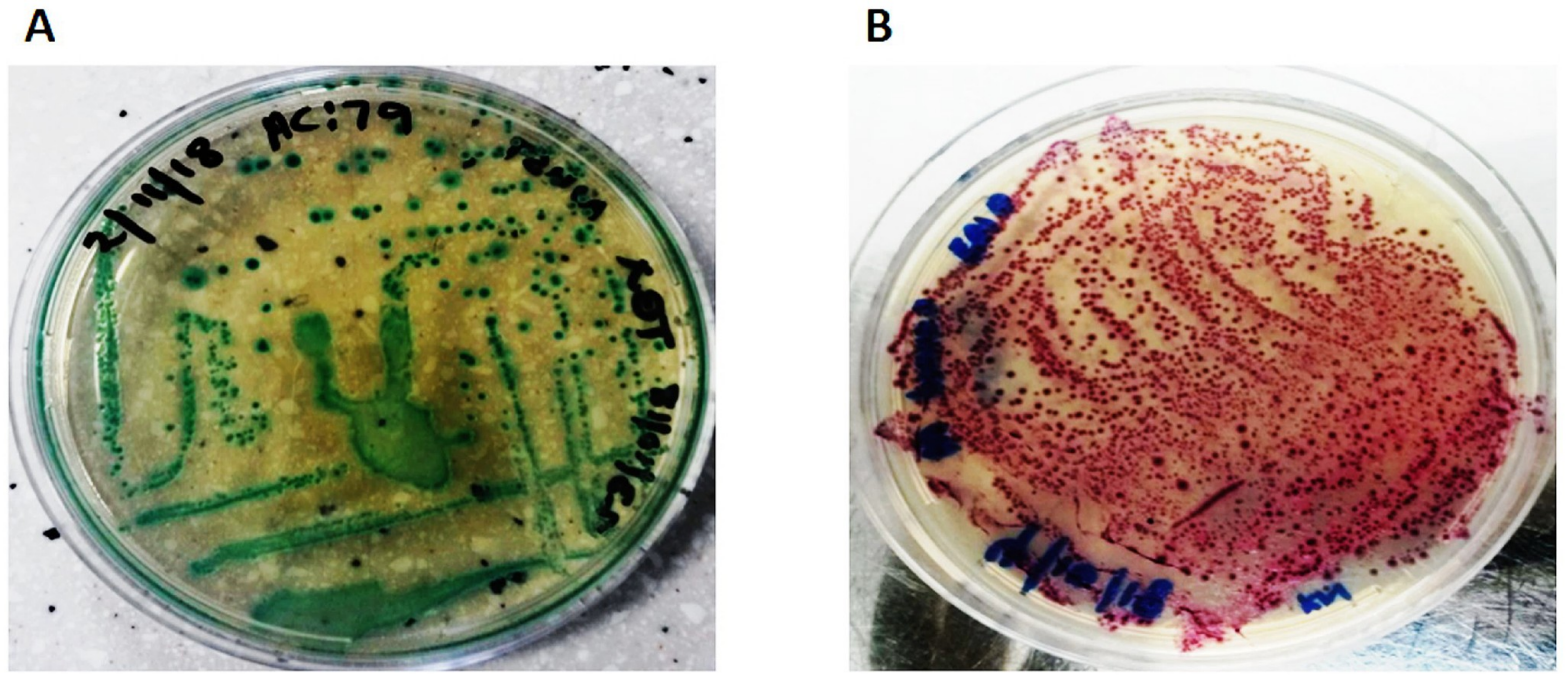
Bacteria culture of *E*. *coli* and STEC from both informal and commercial abattoirs. (**A**) *E*. *coli* cultures appearing blue-green on TBX plate. (**B**) STEC cultures showing typical mauve colonies on CHROMagar STEC plate.

A total of 204 meat samples were investigated from both the informal and commercial abattoirs. *E*. *coli* colonies appeared blue green on TBX plates and STEC cultures appeared mauve on CHROMagar STEC plates (Fig 2).

**Fig 3:**
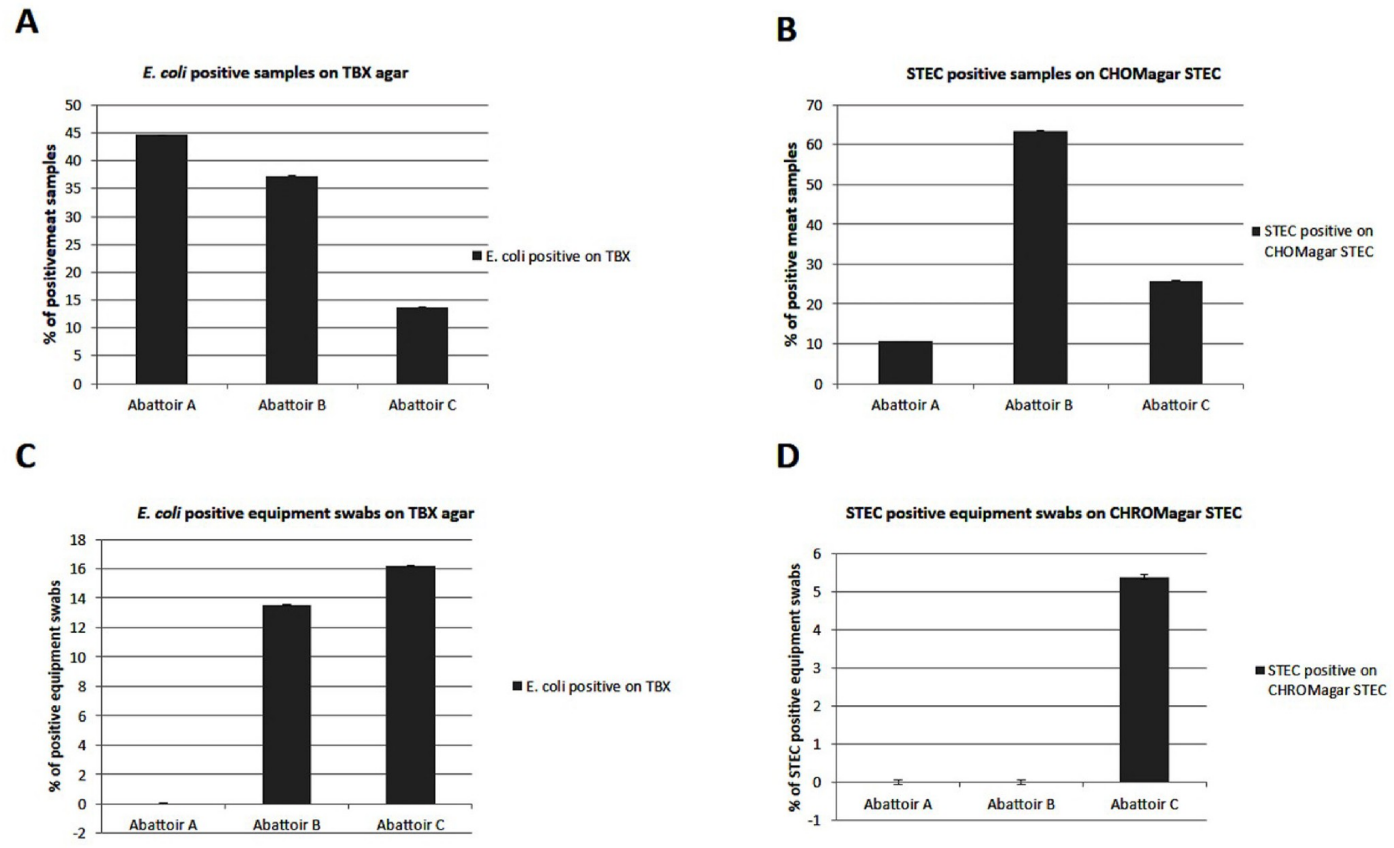
Presumptive *E*. *coli* and STEC positive samples based on growth on TBX and CHROMagar. (**A**) Illustrating 44.6% of samples from abattoir A, 37.25% from abattoir B and 13.72% from abattoir C that showed *E*.*coli* growth on TBX. (**B**) Illustrating 10.58% of meat samples from abattoir A, 63.53% from abattoir B and 25.88% from abattoir C that showed STEC cultures (mauve) on CHROMagar STEC. (**C**) Indicating the presence of *E*.*coli* in 13.51% equipment swabs from abattoir B and 16.21% equipment swabs from abattoir C. (**D**) showing the presence of STEC in 5.40% equipment swabs from abattoir C.

Of the 204 meat samples which were investigated for STEC, the overall prevalence of this pathogen was 85 (41.66%). The meat samples’ results with regard to TBX plates for *E*. *coli* cultures were 91 (44.6%) abattoir A, 76 (37.25%) abattoir B and 28 (13.72%) abattoir C. On the other hand, the meat samples’ results for STEC cultures on CHROMagar STEC plate were 9 (10.58%) abattoir A, 54 (63.53%) abattoir B and 22 (25.88%) abattoir C (Fig 3A and 3B). A total of ten colonies per sample were screened and confirmed.

All the meat samples that were positive on both TBX (*E*. *coli*) and CHROMagar (STEC) gave a 99.9% confirmation when subjected to biochemical test, using VITEK 2 cards. These were then confirmed through biochemical testing and screened for STEC virulence genes intimin (*eae*) and shiga toxin (*stx*) using PCR and STEC primers.

According to the PCR results, *eae* was detected in 8 (8%) out of 100 meat samples from abattoir A, 54 (71.05%) out of 76 meat samples from abattoir B and 22 (78.57%) out of 28 samples from abattoir C. Also, shiga toxin type 1 (*stx*_*1*_*)* was detected in 9 (9%) out of 100 samples from abattoir A, 52 (65.79%) out of 76 samples from abattoir B and 22 (78.57%) out of 28 samples from abattoir C, while shiga toxin type 2 (*stx*_*2*_) was only confirmed in 5 (5%) out of 100 meat samples from Abattoir A and 7 (9. 21%) out of 76 meat samples from abattoir B.

Of all the samples (n = 85) tested by PCR, both eae and *stx*_*1*_ genes were detected in 4 samples from abattoir A, 47 samples from abattoir B and 22 samples from abattoir C. Also, both eae, *stx*_*1*_ and *stx*_*2*_ genes were detected from 4 samples from abattoir A and 5 samples from abattoir B. In addition, 2 samples from abattoir B were all positive for eae and *stx*_*2*_ only. While 1 sample from abattoir A was positive for both *stx*_*1*_ and *stx*_*2*_ genes (Table 3).

**Table 3:** Summary of detected STEC virulence genes from the meat sample bacterial isolates from informal and commercial abattoirs.

STEC virulence gene(s)	No. of positive meat sample bacterial isolates		
	Abattoir A	Abattoir B	Abattoir C
*eae*, *stx*_*1*_	4	47	22
*eae*, *stx*_*2*_	0	2	0
eae, *stx*_*1*_, *stx*_*2*_	4	5	0
*stx*_*1*_, *stx*_*2*_	1	0	0

### Sources of contamination of raw beef by STEC

With regard to equipment swabs (n = 37) collected, 11 (29.73%) showed the presence of *E*. *coli* on TBX plate. These were from informal abattoirs, 5 from abattoir B and 6 from abattoir C. While, from the *CHROMagar* STEC, only 2 (5.40%) out of 37 equipment swabs were STEC positive which were part of the 7 equipment swabs collected from one of the informal abattoirs (abattoir C). All equipment swabs collected from abattoir A (23 swabs) and abattoir B (7 swabs) tested STEC negative (Fig 3C and 3D).

Confirmation of the two positive equipment swabs from abattoir C, to establish the presence of *E*. *coli* in the samples was done by biochemical test using VITEK 2 cards, and confirmed by PCR for stx and eae.

### Proportion of STEC strains in raw beef

Among all eae and stx STEC positive isolates from meat samples (n = 85), only 25 (29.41%) contained one of the major serotype STEC strains (O26, O45, O103, O111, O121, O145 and *E*. *coli* O157:H7, and confirmed by PCR) while 60 (70.58% STEC positive samples) did not contain the target stains. *E*. *coli* O157:H7 was detected from 5 (20%) from abattoir A and 7 (28%) from abattoir B. STEC strain O26 was isolated from 1 (4%) sample from abattoir A, 3 (12%) from abattoir B and 1 (4%) from abattoir C, while STECs O45 was detected in 2 (8%) from abattoir B and 2 (8%) from abattoir C. Also, O103 strain was detected in 1 (4%) from abattoir A, O145 strain was detected in 1 (4%) from abattoir C, while both O45, O111, O121 strains were isolated from 1 (4%) from abattoir A. Another two STEC strains, O45 and O111 were also detected in 1 (4%) from abattoir C. Of the 25 STECs positive samples, each of the four *E*. *coli* O157:H7 positive samples from abattoir A and five *E*. *coli* O157:H7 samples from abattoir B contained both eae, stx_1_, and *stx*_*2*_ virulence genes, while abattoir C only contained positive samples with eae and stx_1_ only (Table 4).

**Table 4:** Characterisation of STECs from meat sample bacterial isolates from both abattoirs.

Type of abattoir	Abattoir name	Strains	No. of samples (n = 25)	% of positive samples	STECs Virulence gene
Commercial	Abattoir A	O26	1	4	*eae*, *stx*_*1*_
abattoir		O103	1	4	*eae*, *stx*_*1*_
		O45, O111, O121	1	4	*eae*, *stx*_*1*_
		O157:H7	4	16	*eae*, *stx*_*1*_, *stx*_*2*_
			1	4	*stx*_*1*_ *stx*_*2*_
Informal	Abattoir B	O26	3	12	*eae*, *stx*_*1*_
abattoirs		O45	2	8	*eae*, *stx*_*1*_
		O157:H7	5	20	*eae*, *stx*_*1*_ *stx*_*2*_
			2	8	*eae*, *stx*_*2*_
	Abattoir C	O26	1	4	*eae*, *stx*_*1*_
		O45	2	8	*eae*, *stx*_*1*_
		O145	1	4	*eae*, *stx*_*1*_
		O45, O111	1	4	*eae*, *stx*_*1*_

## Discussion

STEC represent the significant group of *E*. *coli* that is defined by zoonotic origin [25]. In most cases, human sickness resulting from STEC infection have been related to bovines and their beef products [9]. This study investigated the presence of STEC in raw beef from two informal and one commercial abattoirs in Windhoek, Namibia.

The study showed the presence of STEC virulence genes, shiga toxin (*stx*_*1*_ *and stx*_*2*_) and *eae* in 41.66% raw beef isolates (85/204 beef samples) which was higher than 36.1% (91/252) STEC positive samples reported by Llorente *et al* [4], but lower than 66% STEC positive raw beef samples reported in a Bangladesh study [25]. A study conducted by Llorente et., [4] showed the presence of *stx*_*1*_ *and stx*_*2*_ 5.3% (3/57) and *eae* 26.3% (15/57) virulence genes in samples that was low in relation with the current study findings where 40.68% samples (83 of 85 STEC positive samples) contained *stx*_*1*_ 5.88% contained *stx*_*2*_ *and* 98.82*%* (84 STEC positive samples) contained *eae* virulence gene. The high percentage (98.82%) of eae-positive STEC strains could be as a consequence of the culture medium used (CHROMagar). CHROMagar allows the isolation of STEC having the *ter* gene cassette, which confer resistance to tellurite. The *ter* gene cluster is significantly correlated with the presence of the *eae* gene [26]. Therefore, it is reasonable that the STEC strains isolated on CHROMagar were mostly *eae*-positive. Recently, it has been reported that the burden of disease caused by *eae*-negative STEC strains has increased in several countries [27, 28]. Therefore, a major limitation of this study is the use of CHROMagar as selective media for STEC, in which *eae*-negative STEC strains of clinical importance (O91 and O113 serogroups) are not detected [26].

Importantly, this study findings on beef samples (9% STEC positive samples) from commercial abattoirs correlates with a previous study by Arthur *et al* [29] that showed 10.1% STEC positive samples in a commercial beef processing plant. In addition to this, the current study findings closely corresponds with the results of Oloyede *et al* [30] who identified STEC in 6.7% (8/120) raw beef samples collected from abattoirs in Abeokuta, Southwest Nigeria. The study has further observed a very high number of STEC positive meat samples (73.07%, 76/104 samples) from informal abattoirs (abattoir B and C) compared to 1.7% positive samples (37/2100 beef carcass swabs) observed in a study conducted by Mwai [31] at three slaughterhouses in Nairobi. Similarly, insufficient slaughter process, lack of control on continuous movement of production men from dirt to clean area and from clean to dirty area, and poor hygiene practices during de-hiding of beef carcasses might also have influenced the high number of STEC positive results at informal abattoirs. However, aspects such as different geographical location, sampling method and isolation procedures make it hard to compare findings of different research studies [32].

Informal abattoirs are regarded as low throughput slaughterhouses of which some are not registered and often contribute to illegal slaughtering of animals. These abattoirs are characterised by multiple failure in the slaughter process with inadequate infrastructure, deficiency of proper hygiene, lack of sanitation and standard operating procedures (SSOPs) and lack of post-mortem and ante-mortem inspection, frequently resulting in meat contamination by microorganisms. On the other hand, Commercial abattoirs are registered slaughterhouses that are characterised by more advanced slaughter processes and slaughter equipment, with high level of hygiene practices, professionally trained production employees and documented standard operating procedures. There is competent authority personnel employed at such abattoirs, high level of process control (traceability system) from live cattle receiving point to the destination of finished meat product, and they produce meat for both local and export market [33]. According to the study findings, there was high level of contamination (73.07%) of beef by STEC at informal abattoirs than at commercial abattoirs (9%) in Windhoek. These might be a result of poor hygienic practices and insanitary dressing procedures, where carcasses get contaminated during the dressing process through cross contamination (by STEC pathogen) from the hide (of unskinned carcass, via contact with skinned carcass) and from infected ingesta and faecal material during evisceration.

Although a number of studies have observed that faecal infected cattle hide and infected ingesta plays a role in the contamination of carcasses by STEC, with equipment (knives and saws) being vectors [8] and hands of operators being vehicle of contamination [34], there is no correlation between these previous reports and the findings of this study since all hand swabs collected were STEC negative, and there were only 5.40% (2/37 equipment swabs) STEC positive equipment swabs. The negative hand swab results imply that there is a lack of association between the STEC positive meat samples and the hands of beef carcass handlers from all abattoirs or the methods used for detection were not sensitive enough. In this case future studies sampling faeces, hides, and carcasses during processing for STEC would be followed-up in trying to identify the sources of STEC.

In contrast to findings by Svoboda *et al* [35], that had a larger number of environmental samples (55.5%, 151/272) having STEC i.e. 4.0% (*eae)* and 7.7% (*stx*_*1*_ and *stx*_*2*_) virulence genes, our current study only showed 4.50% having *eae* and *stx*_*1*_. Also, Brusa *et al* [36] reported the prevalence of STEC on environmental samples (37.5%, 6 of 16 samples) collected from a beef retail market (which served as slaughterhouse and market at the same time) in Argentina. With regard to this study findings, the 2 STEC positive equipment (skinning knives, from informal abattoir C) might have contributed to beef carcass contamination by STEC at abattoir C which had the highest number of STEC positive meat samples (78.57%, 22 of 28 samples) compared to abattoir A and B. Since the study observed the presence of STEC on only 2 equipment swabs (low number of sample, which has been collected from skinning knives), it is therefore speculated that the knives might have been contaminated with infected bacteria from the hide during carcass dressing process and that the operators failed to clean them properly. Although this association could not be ascertained in this study thus, future studies entailing bacterial tracing methods (e.g. Multilocus sequencing typing, restriction fragment length polymorphism analysis (RFLP), or whole-genome sequencing analysis) will be employed. On the other hand, the low number of positive equipment may be due to the reason that other equipment were not exposed to infected material (like faecal contamination on the hide), or due to the fact that asymptomatic cattle are the main carrier of STEC and contamination mostly occur through cross contamination with the hide, faecal or ingesta [37].

Nevertheless, the study findings indicates that the incidences of STEC strains (STECs) in raw beef from informal and commercial abattoirs in Windhoek were higher (29.41%, 25/85 STEC positive samples) than those (17.64%, 136/771 STEC positive samples) reported by Molini *et al* [3]. In the study conducted at one local abattoir in Windhoek, 87 positive samples contained one of the top 6 non-O157 STECs (O26, O45, O103, O111, O121 and O145 strains) while 35 samples of 136 positive samples were found positive for more than one STEC, whereas in the current study 29.41%, 25/85 STEC positive samples contained the major STECs (O26, O45, O103, O111, O121, O145 and *E*. *coli* O157:H7) with 11 samples of 25 STECs positive samples containing one of the top 6 non O157 STECs. The study findings show that *E*. *coli* O157:H7 strain was dominant among all the positive meat samples followed by O26 STEC strains which was high among the major targeted non O157 STECs, corresponding with the findings of Molini *et al* [3] whose study showed 6.61%, 9/136 STEC positive meat samples being O26 strain, higher than all the high top 6 STECs isolated. Unlike the results of the present study, Molini et al. [3] results were not confirmed from the isolated colonies.

The findings of the present study have shown a clear evidence of the presence of STEC high virulence gene (*stx*_*2*_) in high number of beef sample from informal abattoirs, 28% (7/25) than from commercial abattoirs (20%, 5/25 STEC positive samples). Hence the 7 samples that were positive for *stx*_*2*_ from informal abattoir were also positive for *E*. *coli* O157:H7, corresponding with the findings by Oloyede *et al* [30] who reported that *stx*_*2*_ virulence gene was isolated from 87.5%,7/8 *E*. *coli* O157:H7 positive samples of 120 raw beef samples collected from abattoirs in Abeokuta, Southwest Nigeria.

From the results, it is clear that STEC virulence genes, *eae*, *stx*_*1*_ and *stx*_*2*_ (high virulence gene) and the major STEC strains (O26, O45, O103, O111, O121, O145 and O157:H7) are present in meat samples of both the informal and commercial abattoirs albeit at lower levels in commercial abattoirs. Although one sample of O157:H7 from abattoir A lacked the eae gene, although this is uncommon it has been previously observed in other studies [38–40]. This has revealed the risk and exposure of beef consumers to STEC infections such as diarrhoea, haemorrhagic colitis (HC) and HUS which in severe cases leads to lethality. Therefore, these findings necessitate the development and implementation of effective measures for the prevention of foodborne infections (caused by STEC) and elimination of beef contamination by STEC. Thus, further studies should be geared towards establishing the sources and route of beef carcass contamination by STEC strains and to investigate the association between STEC positive meat samples and equipment swabs collected from the same abattoirs.

## Supporting information

S1 Rawdata (RAR)

## References

[1] Majowicz SE, Scallan E, Jones-Bitton A, Sargeant JM, Stapleton J, Angulo FJ, et al Global incidence of human Shiga toxin–producing Escherichia coli infections and deaths: a systematic review and knowledge synthesis. Foodborne Pathog Dis. 2014;11(6):447–55. 10.1089/fpd.2013.1704 24750096 PMC4607253

[2] Hedican EB, Medus C, Besser JM, Juni BA, Koziol B, Taylor C, et al Characteristics of O157 versus non-O157 Shiga toxin-producing Escherichia coli infections in Minnesota, 2000–2006. Clin Infect Dis. 2009;49(3):358–64. 10.1086/600302 19548834

[3] Molini U, Khaiseb S, Kamwi J. Detection of non O157: H7 Shiga toxin-producing Escherichia coli (STEC) serogroups O26, O45, O103, O111, O121 and O145 from beef trim in Namibia. Biological and Chemical Research. 2016;7.

[4] Llorente P, Barnech L, Irino K, Rumi MV, Bentancor A. Characterization of Shiga toxin-producing Escherichia coli isolated from ground beef collected in different socioeconomic strata markets in Buenos Aires, Argentina. BioMed research international. 2014;2014 10.1155/2014/795104 25006586 PMC4070525

[5] Brooks JT, Sowers EG, Wells JG, Greene KD, Griffin PM, Hoekstra RM, et al Non-O157 Shiga toxin–producing Escherichia coli infections in the United States, 1983–2002. The Journal of infectious diseases. 2005;192(8):1422–9. 10.1086/466536 16170761

[6] Bettelheim K. Role of non-O157 VTEC. J Appl Microbiol. 2000;88(S1):38S–50S. 10.1111/j.1365-2672.2000.tb05331.x 10880178

[7] Blanco M, Blanco JE, Blanco J, Mora A, Prado C, Alonso MP, et al Distribution and characterization of faecal verotoxin-producing Escherichia coli (VTEC) isolated from healthy cattle. Vet Microbiol. 1997;54(3–4):309–19. 10.1016/s0378-1135(96)01292-8 9100331

[8] Hernández M, Hansen F, Cook N, Rodríguez-Lázaro D. Real-time PCR methods for detection of foodborne bacterial pathogens in meat and meat products Safety of Meat and Processed Meat: Springer; 2009 p. 427–46.

[9] Hussein H. Prevalence and pathogenicity of Shiga toxin-producing Escherichia coli in beef cattle and their products. J Anim Sci. 2007;85(suppl_13):E63–E72. 10.2527/jas.2006-421 17060419

[10] Bollinger LM. Effects of season and animal factors on prevalence of Shigatoxin-producing Escherichia coli in beef cattle: University of Nevada, Reno; 2004.

[11] Control CFD, Prevention. Two multistate outbreaks of Shiga toxin--producing Escherichia coli infections linked to beef from a single slaughter facility-United States, 2008. MMWR Morbidity and mortality weekly report. 2010;59(18):557 20467414

[12] Effler E, Isaäcson M, Arntzen L, Heenan R, Canter P, Barrett T, et al Factors contributing to the emergence of Escherichia coli O157 in Africa. Emerg Infect Dis. 2001;7(5):812 10.3201/eid0705.017507 11747693 PMC2631888

[13] Koyange L, Ollivier G, Muyembe J-J, Kebela B, Gouali M, Germani Y. Enterohemorrhagic Escherichia coli O157, Kinshasa. Emerg Infect Dis. 2004;10(5):968 10.3201/eid1005.031034 15216857 PMC3323201

[14] Tau NP, Meidany P, Smith AM, Sooka A, Keddy KH. Escherichia coli O104 associated with human diarrhea, South Africa, 2004–2011. Emerg Infect Dis. 2012;18(8):1314 10.3201/eid1808.111616 22840375 PMC3414021

[15] Hassanien A, Aidaros H, Talaat M, Nouman T, El-Mossalami E. Contamination of beef carcasses during slaughtering in two Egyptian slaughterhouses.

[16] Francis M, Dziva F, Mlambo C, Akpaka P. Shiga Toxin Producing Escherichia coli (STEC) in Food Producing Animals from Trinidad and Tobago. Journal of Experimental Agriculture International. 2016:1–9.

[17] Kothari CR. Research methodology: Methods and techniques: New Age International; 2004.

[18] Chapter I. FSIS DIRECTIVE 10,010.1 Rev. 3 Verification Activities for Escherichia coli O157: H7 in Raw Beef Products.

[19] Rigobelo E, de Ávila F. Shiga toxin-Producing Escherichia coli from beef carcass. J Microbiol Res. 2012;2:103–7.

[20] Bai J, Shi X, Nagaraja T. A multiplex PCR procedure for the detection of six major virulence genes in Escherichia coli O157: H7. J Microbiol Methods. 2010;82(1):85–9. 10.1016/j.mimet.2010.05.003 20472005

[21] Bai J, Paddock ZD, Shi X, Li S, An B, Nagaraja TG. Applicability of a multiplex PCR to detect the seven major Shiga toxin–producing Escherichia coli based on genes that code for serogroup-specific O-antigens and major virulence factors in cattle feces. Foodborne Pathog Dis. 2012;9(6):541–8. 10.1089/fpd.2011.1082 22568751

[22] Sarimehmetoglu B, Aksoy MH, Ayaz ND, Ayaz Y, Kuplulu O, Kaplan YZ. Detection of Escherichia coli O157: H7 in ground beef using immunomagnetic separation and multiplex PCR. Food Control. 2009;20(4):357–61.

[23] Bertrand R, Roig B. Evaluation of enrichment-free PCR-based detection on the rfbE gene of Escherichia coli O157—application to municipal wastewater. Water research. 2007;41(6):1280–6. 10.1016/j.watres.2006.11.027 17222887

[24] Fleiss J, Levin B, Paik M. Statistical methods for rates and proportions. John Wiley & Sons New York 1981;870.

[25] Islam MA, Mondol AS, Azmi IJ, de Boer E, Beumer RR, Zwietering MH, et al Occurrence and characterization of Shiga toxin–producing Escherichia coli in raw meat, raw milk, and street vended juices in Bangladesh. Foodborne Pathog Dis. 2010;7(11):1381–5. 10.1089/fpd.2010.0569 20704491

[26] Jenkins C, Perry NT, Godbole G, Gharbia S. Evaluation of chromogenic selective agar (CHROMagar STEC) for the direct detection of Shiga toxin-producing Escherichia coli from faecal specimens. J Med Microbiol. 2020;69(3):487–91. 10.1099/jmm.0.001136 31935188 PMC7431092

[27] Montero DA, Velasco J, Del Canto F, Puente JL, Padola NL, Rasko DA, et al Locus of adhesion and autoaggregation (LAA), a pathogenicity island present in emerging Shiga toxin–producing Escherichia coli strains. Sci Rep. 2017;7(1):1–13. 28765569 10.1038/s41598-017-06999-y PMC5539235

[28] Montero DA, Canto FD, Velasco J, Colello R, Padola NL, Salazar JC, et al Cumulative acquisition of pathogenicity islands has shaped virulence potential and contributed to the emergence of LEE-negative Shiga toxin-producing Escherichia coli strains. Emerging Microbes & Infections. 2019;8(1):486–502. 10.1080/22221751.2019.1595985 30924410 PMC6455142

[29] Arthur TM, Barkocy-Gallagher GA, Rivera-Betancourt M, Koohmaraie M. Prevalence and characterization of non-O157 Shiga toxin-producing Escherichia coli on carcasses in commercial beef cattle processing plants. Appl Environ Microbiol. 2002;68(10):4847–52. 10.1128/aem.68.10.4847-4852.2002 12324330 PMC126400

[30] Oloyede A, Afolabi O, Olalowo O. Molecular detection of virulence genes and antibiotic resistance patterns of Escherichia coli O157: H7 isolated from raw beef sold in Abeokuta, South-West Nigeria. Nigerian Journal of Biotechnology. 2016;31(1):15–21.

[31] Mwai C. Risk of contamination of beef carcasses with Escherichia coli O157: H7 from slaughterhouses in Nairobi, Kenya: University of Nairobi; 2011.

[32] Xia X, Meng J, McDermott PF, Ayers S, Blickenstaff K, Tran T-T, et al Presence and characterization of Shiga toxin-producing Escherichia coli and other potentially diarrheagenic E. coli strains in retail meats. Appl Environ Microbiol. 2010;76(6):1709–17. 10.1128/AEM.01968-09 20080990 PMC2837998

[33] Cook EAJ, de Glanville WA, Thomas LF, Kariuki S, de Clare Bronsvoort BM, Fèvre EM. Working conditions and public health risks in slaughterhouses in western Kenya. BMC Public Health. 2017;17(1):14 10.1186/s12889-016-3923-y 28056885 PMC5217581

[34] McEvoy J, Doherty A, Finnerty M, Sheridan J, McGuire L, Blair I, et al The relationship between hide cleanliness and bacterial numbers on beef carcasses at a commercial abattoir. Lett Appl Microbiol. 2000;30(5):390–5. 10.1046/j.1472-765x.2000.00739.x 10792669

[35] Svoboda AL, Dudley EG, DebRoy C, Mills EW, Cutter CN. Presence of Shiga toxin–producing Escherichia coli O-groups in small and very-small beef-processing plants and resulting ground beef detected by a multiplex polymerase chain reaction assay. Foodborne Pathog Dis. 2013;10(9):789–95. 10.1089/fpd.2012.1445 23742295

[36] Brusa V, Aliverti V, Aliverti F, Ortega EE, de la Torre JH, Linares LH, et al Shiga toxin-producing Escherichia coli in beef retail markets from Argentina. Frontiers in Cellular and Infection Microbiology. 2013;2:171 10.3389/fcimb.2012.00171 23346554 PMC3548221

[37] Baumgartner A, Niederhauser I, Diston D, Moor D. Enterohemorrhagic Escherichia coli (EHEC) in water from karst springs: detection with real-time PCR and isolation of strains. Journal für Verbraucherschutz und Lebensmittelsicherheit. 2016;11(4):353–7.

[38] Woodward MJ, Best A, Sprigings KA, Pearson GR, Skuse AM, Wales A, et al Non-toxigenic Escherichia coli O157: H7 strain NCTC12900 causes attaching-effacing lesions and eae-dependent persistence in weaned sheep. Int J Med Microbiol. 2003;293(4):299–308. 10.1078/1438-4221-00264 14503794

[39] McKee ML, Melton-Celsa AR, Moxley RA, Francis DH, O’Brien AD. Enterohemorrhagic Escherichia coli O157: H7 requires intimin to colonize the gnotobiotic pig intestine and to adhere to HEp-2 cells. Infect Immun. 1995;63(9):3739–44. 10.1128/IAI.63.9.3739-3744.1995 7642319 PMC173523

[40] Dean-Nystrom EA, Bosworth BT, Moon HW, O’brien AD. Escherichia coli O157: H7 requires intimin for enteropathogenicity in calves. Infect Immun. 1998;66(9):4560–3. 9712821 10.1128/iai.66.9.4560-4563.1998 PMC108559

